# Development and Calibration of the PREMIUM Item Bank for Measuring Respect and Dignity for Patients with Severe Mental Illness

**DOI:** 10.3390/jcm11061644

**Published:** 2022-03-16

**Authors:** Sara Fernandes, Guillaume Fond, Xavier Zendjidjian, Pierre Michel, Karine Baumstarck, Christophe Lançon, Ludovic Samalin, Pierre-Michel Llorca, Magali Coldefy, Pascal Auquier, Laurent Boyer, Collaborators of the French PREMIUM Group

**Affiliations:** 1 CEReSS-Health Service Research and Quality of Life Center (UR3279), Aix-Marseille University, 13005 Marseille, France; guillaume.fond@gmail.com (G.F.); xavier.zendjidjian@ap-hm.fr (X.Z.); karine.baumstarck@ap-hm.fr (K.B.); christophe.lancon@ap-hm.fr (C.L.); pascal.auquier@univ-amu.fr (P.A.); laurent.boyer@ap-hm.fr (L.B.); 2 Fondation FondaMental, 94000 Créteil, France; lsamalin@chu-clermontferrand.fr (L.S.); pmllorca@chu-clermontferrand.fr (P.-M.L.); 3 Centre National de la Recherche Scientifique (CNRS), Aix-Marseille School of Economics (AMSE), Aix-Marseille University, 13005 Marseille, France; pierre.michel@univ-amu.fr; 4 Institut de Recherche et Documentation en Economie de la Santé (IRDES), 75019 Paris, France; coldefy@irdes.fr

**Keywords:** psychiatry, mental health, schizophrenia, depressive disorders, bipolar disorders, patient-reported experience measures, health services research

## Abstract

Most patient-reported experience measures (PREMs) are paper-based, leading to a high burden for patients and care providers. The aim of this study was to (1) calibrate an item bank to measure patients’ experience of respect and dignity for adult patients with serious mental illnesses and (2) develop computerized adaptive testing (CAT) to improve the use of this PREM in routine practice. Patients with schizophrenia, bipolar disorder, and major depressive disorder were enrolled in this multicenter and cross-sectional study. Psychometric analyses were based on classical test and item response theories and included evaluations of unidimensionality, local independence, and monotonicity; calibration and evaluation of model fit; analyses of differential item functioning (DIF); testing of external validity; and finally, CAT development. A total of 458 patients participated in the study. Of the 24 items, 2 highly inter-correlated items were deleted. Factor analysis showed that the remaining items met the unidimensional assumption (RMSEA = 0.054, CFI = 0.988, TLI = 0.986). DIF analyses revealed no biases by sex, age, care setting, or diagnosis. External validity testing has generally supported our assumptions. CAT showed satisfactory accuracy and precision. This work provides a more accurate and flexible measure of patients’ experience of respect and dignity than that obtained from standard questionnaires.

## 1. Introduction

Severe mental illnesses (SMIs), including schizophrenia, bipolar disorder, and major depressive disorder, are associated with suboptimal quality of care that seems to worsen over time [1,2,3]. These illnesses are often unrecognized or misdiagnosed, leading first to a prolonged duration of untreated illness [4,5,6], increased risk of relapse and hospitalization [7,8,9], and subsequently, to poorer outcomes in treatment response, symptoms, and quality of life [10,11]. Care access is crucial for SMIs patients [12], and quality of care plays a major role in the chances to reach full-functional recovery [13,14]. It is therefore essential to measure the quality of care to identify areas in which changes are needed. The patient’s perspective is now considered to be an important measure of the quality of care, and the use of patient-reported experience measures (PREMs) is recommended by many organizations worldwide [15]. Most PREMs are paper-based, are frequently too lengthy, and have fixed content, leading to a high burden on both patients and care providers, making such PREMs difficult to use in routine clinical practice [16]. Modern statistical methods based on item response theory (IRT) are used to develop item banks and computerized adaptive testing (CAT) to overcome some of these limitations [17,18,19]. In an item bank, since the items are calibrated by an IRT model, the scores resulting from the administration of different subsets of items can be compared with one another. These item banks can then be used to develop static short forms or CATs [17]. CATs allow administering only the most informative items for a given patient, thereby optimizing the precision of the assessment while reducing the length of the questionnaire and completion time [20]. Item banks and CATs have been developed in the field of mental health, but these have focused on health outcomes (e.g., quality of life [21]), and none are available for the experience of SMIs patients in the French psychiatric context. To improve the use of patient-reported measures in the field of mental health, the Patient-Reported Experience Measure for Improving qUality of care in Mental health (PREMIUM) French group is currently developing item banks for PREMs and associated CATs [22]. In our work, we have identified respect and dignity as an important dimension of the experience of adult patients with SMIs [23], as has the work carried out by the PAtient Reported Indicators Survey (PaRIS) working group of the organization for economic cooperation and development (OECD) [24].

The aim of this study was, therefore, to (1) calibrate an item bank to measure patients’ experience of respect and dignity for adult patients with SMIs and (2) develop a CAT to improve the use of this PREM in routine practice.

## 2. Materials and Methods

### 2.1. Study Design and Setting

Data taken from a national, multicenter, cross-sectional study were used. Patients were recruited between January 2016 and November 2020 from inpatient and outpatient departments (including full-time hospitalization, part-time hospitalization, and outpatient care) of the Assistance Publique—Hôpitaux de Marseille, from the FondaMental Foundation’s network of expert centers and through an online survey. All participants gave their informed consent before participating in the study. This study was approved by the competent ethics committee (CPP-Sud Méditerranée V, 12 November 2014, n°2014-A01152-45).

### 2.2. Inclusion Criteria

The inclusion criteria for this study were as follows: a clinical diagnosis of schizophrenia, bipolar disorder, or major depressive disorder according to the DSM-5 [25]; inpatient or outpatient psychiatric care, regardless of current or previous care, duration, or severity of illness; age over 18 years and under 65 years; and ability to read and speak French without comprehension problems. The exclusion criteria of this study were as follows: mental retardation or decompensated organic illness; vulnerable persons (i.e., pregnant or nursing women, persons under legal protection measures, etc.); inability to complete a self-administered questionnaire; and withdrawal of consent.

### 2.3. Data Collection

The following data were collected:

Socio-demographic data: sex; age; educational level; marital status; and occupational status.

Clinical data: main diagnosis (schizophrenia, bipolar disorder or major depressive disorder); duration of illness; psychological, social, and occupational functioning of an individual as measured using the Global Assessment of Functioning scale [26] (GAF, ranging from 0 to 100, with a higher score indicating better functioning); health-related quality of life (QoL) as measured using the medical outcome study 12-item Short Form (SF-12) [27], which describes 8 QoL dimensions: physical functioning (PF), social functioning (SF), role physical (RP), role emotional (RE), mental health (MH), vitality (VT), bodily pain (BP), general health (GH), and 2 composite scores for physical (PCS) and mental (MCS) quality of life (ranging from 0 to 100, with higher scores indicating better quality of life).

The respect and dignity item bank (PREMIUM-RD) includes 24 items as well as an overall satisfaction item (“Overall, you feel you were treated with respect and dignity”) and a corresponding visual analog scale (VAS) (ranging from 0 to 10). All items were scored on a 5-point Likert scale (“strongly disagree”, “disagree”, “neither agree nor disagree”, “agree”, “strongly agree”) with a “not applicable” response option. The coding of negatively worded items was reversed so that higher scores indicated a greater experience of respect and dignity shown by mental health professionals. The assessment period referred to the four weeks prior to administration.

### 2.4. Statistical Analysis

The general steps for the development of the item banks and associated CATs of the PREMIUM project have been described in detail previously [22]. Based on rigorous and well-established methodology [17,28,29], the procedure was divided into four steps: (1) conceptual work and definition of domain mapping; (2) item selection; (3) item bank calibration and CAT simulations; and (4) CAT validation. In this article, we report the main results of the third step for one of the seven PREMIUM item banks [21], which measured respect and dignity (PREMIUM-RD).

#### 2.4.1. Descriptive Analysis

The 24 items of the PREMIUM-RD bank were first subjected to descriptive analysis, and items that presented (1) high missing value rates (>70%); (2) extreme skewness (>95% of response rate in one category or an absolute coefficient > 4); or (3) inter-item correlation coefficients higher than 0.70 were excluded. Internal consistency was evaluated by calculating Cronbach’s alpha coefficient, with α > 0.70 considered to be acceptable [30].

#### 2.4.2. Evaluation of the Assumptions of the IRT Model

Use of an IRT model requires that the key assumptions underlying the IRT framework be fulfilled, including (i) unidimensionality, (ii) local independence, and (iii) monotonicity [19].

The unidimensionality assumption was evaluated based on a 1-factor confirmatory factor analysis (CFA) with the weighted least square mean and variance (WLSMV) estimator due to the ordinal nature of the data [28]. The following indices were used to assess the goodness-of-fit of the model to the data, with an acceptable fit defined by the root mean square error of approximation (RMSEA) ≤ 0.08, the comparative fit index (CFI), and the Tucker–Lewis index (TLI) ≥ 0.95 [31,32]. If the CFA showed poor fit, an exploratory factor analysis (EFA) was performed after randomly dividing the entire sample into 2 subsamples (n = 229 for EFA and n = 229 for CFA). The number of factors to be kept was based on the Kaiser-Guttman’s rule (eigenvalues ≥ 1), differences in the magnitude of eigenvalues between factors (a ratio greater than 4 is expected), the scree test (looking for an “elbow” in the curve), parallel analysis and factor loadings (with minimum item loadings set at 0.40) [28]. Next, to investigate whether item responses are sufficiently unidimensional for IRT application, we used a bifactor model [33]. The bifactor model assumes one general factor (in this case, experience of respect and dignity), onto which all items load, and several group factors, onto which unique subsets of items load [33]. The percentage of explained common variance (ECV) and the omega hierarchical (*ω_h_*/*ω_hs_*) coefficients accounted for by the general factor and by group factors were calculated, with an expected *ω_h_* coefficient for the general factor greater than or equal to 0.70 and the expected percentage of ECV for the general factor greater than or equal to 60% to support unidimensionality [34,35].

Local independence was examined using residual correlations from the final CFA model. All residual correlations greater than 0.20 (or 0.25) indicated possible local dependence, leading to the deletion of the item with the highest residual correlation with other items in the bank [36,37].

Finally, monotonicity was evaluated by visual inspection of item characteristic curves (ICCs), with each response category expected to have a maximum probability of being selected on a specific range of the latent trait continuum. If two categories were not sufficiently discriminative for a particular item, they were collapsed, and the resulting model was re-estimated. The deviations of the Akaike information criterion (AIC) [38] and the Bayes information criterion (BIC) [39] between the final model (recoded items) and the initial model (no recoded items) were computed to ensure that the recoding process resulted in a substantial improvement in the model.

#### 2.4.3. Calibration and Fitting of an IRT Model to the Data

The generalized partial credit model (GPCM) was used to calibrate the responses to the items [40]. The GPCM is appropriate for items with ordered polytomous response options (such as Likert scales). In the GPCM, each item has a discrimination parameter (i.e., the ability to distinguish among individuals with different levels of a latent trait) and a set of threshold parameters (i.e., the item’s difficulty). The GPCM is a generalization of the partial credit model (PCM), in which the discrimination parameter is equal across all items [41]. The likelihood ratio test [42], as well as the information criteria AIC [38] and BIC [39], were calculated and compared to select the IRT model that best fit the data. The item parameters (discrimination and thresholds) were then estimated under the selected model.

Item parameters were estimated using the maximum marginal likelihood estimation (MMLE) implemented via the expectation–maximization (EM) algorithm [43]. Items with a discrimination parameter below 0.50 were also considered problematic [44,45], as they were not sufficiently informative and were thus removed from the item bank. Next, the goodness-of-fit was evaluated by computing the infit mean square (Infit MnSq) statistic [46], with an expected value in the range [0.7–1.3] [47].

#### 2.4.4. Evaluation of Differential Item Functioning (DIF)

Differential item functioning (DIF) analyses were carried out to see if all items in the PREMIUM-RD bank in the same way across different subgroups [48,49], identified by sex (men vs. women), age (median split: patients 37 years or younger vs. patients older than 37 years), care setting (outpatient vs. inpatient), and psychiatric diagnosis (schizophrenia vs. bipolar disorder vs. major depressive disorder). If an overall DIF was detected at a level of *p* < 0.01, the magnitude was assessed according to Zumbo’s DIF classification by computing the pseudo R^2^ change (ΔR^2^):negligible if ΔR^2^ < 0.13, moderate if 0.13 < ΔR^2^ < 0.26, and large if ΔR^2^ > 0.26 [50]. Items with a large DIF were excluded from the item bank.

Latent trait scores (θ) for each respondent were estimated by Bayesian expected a posteriori (EAP) estimation [51]. Then, a linear transformation was performed to have θ scores ranging from 0 to 100 (the higher the score was, the better the experience of respect and dignity). Item and test information were also calculated.

#### 2.4.5. External Validation of the Item Bank

External validity was examined by hypothesizing that PREMIUM-RD scores should be positively and moderately correlated with GAF scores and SF-12 dimension scores, but also positively and strongly correlated with scores on the overall satisfaction item and the corresponding VAS. Discriminant validity was examined by testing the association of PREMIUM-RD scores with socio-demographic (i.e., age, sex, educational level, marital status, and employment status) and clinical (i.e., care setting, duration of illness, and main diagnosis) characteristics using t-tests, analysis of variance (ANOVA), and Pearson’s correlation coefficients.

#### 2.4.6. Elaboration of Item Administration algorithm

CAT simulations were performed using both real response data (i.e., complete response patterns to items in the final PREMIUM-RD item bank) and simulated data (i.e., after imputation of plausible missing responses using IRT-based estimation).

The CAT algorithm began by selecting the starting item based on the maximum Fisher information (MFI) criterion, which is relevant to polytomous items and adapted to a unidimensional item bank [52]. Based on the response to this item, an initial latent trait estimate (θ) was computed using the EAP estimate [51]. The CAT algorithm then selected as the next item the item with the highest information for the current θ estimate. The θ estimate was iteratively re-estimated based on the responses to previous items using the EAP estimate. Finally, the CAT algorithm ended when the stopping rule used was reached, which corresponded to the prespecified level of measurement precision based on the standard error of measurement (SEM) [53]. An acceptable range was defined as 0.33 to 0.55, corresponding to reliability coefficients between 0.90 and 0.70 [53]. Three scenarios with different stopping rules corresponding to SEM values of 0.33, 0.44, and 0.55 were simulated and compared using the following accuracy and precision indicators: correlation coefficients (r) between CAT scores and scores based on the full set of items in the bank with expected values greater than or equal to 0.90 and the root mean square error (RMSE) with expected values less than or equal to 0.30 [54].

All of the statistical analyses were performed using the following software: IBM PASW SPSS version 20.0 [55], MPlus version 7.0 [56], and R version 4.0.5 [57], using packages “mirt” [58], “lordif” [59], “BifactorIndicesCalculator” [60], and “mirtCAT” [61].

## 3. Results

### 3.1. Description of the Cohort

The sample included 458 SMIs patients; the majority of the patients were men (61%), single (76%), with an education level of bachelor’s degree or higher (68%), and unemployed (75%). Most of them were outpatients (84%), and among the inpatients (16%), 30% were under constraint. The mean age was 38.1 years (SD ± 12.0). Approximately 65% of patients had a main diagnosis of schizophrenia, while 20% and 15% had a diagnosis of bipolar disorder or major depressive disorder, respectively. The mean duration of illness was 12.3 years (SD ± 8.6). The socio-demographic and clinical characteristics of the sample are presented in Table 1.

### 3.2. Descriptive Analysis

For the initial 24-item pool, the mean ranged from 2.36 ± 1.26 to 3.50 ± 0.82. The floor and ceiling effects ranged from 0.4 to 7.2% and from 15.1 to 60.0%, respectively. Each item had an acceptable skewness coefficient (ranging from −2.15 to −0.35), and missing values ranged from 0.0% to 30.8%. Inter-item correlation coefficients ranged from 0.16 to 0.78 (all with *p* < 0.001, data not shown). Following this step, items 5, 15, and 17 were discarded from the item bank because they exhibited inter-items correlations that were too high (>0.72), reflecting redundancy between items. These characteristics are presented in Table 2.

### 3.3. Evaluation of the Assumptions of an IRT Model

The fit indices of the one-factor CFA model were not adequate (RMSEA = 0.106, 95% CI [0.097–0.115], CFI = 0.942 and TLI = 0.935). In the EFA, the eigenvalue of the first factor was 12.0, and the eigenvalue of the second factor was 1.9. The ratio between the first and second eigenvalues was 6.3, and the total amount of variance explained by the first factor was 57.3%. The scree plot and parallel analysis revealed two predominant factors, and all items loaded suitably on the first factor (>0.40). Additionally, 17 of the 21 items in the bank were recoded after examining the item characteristic curves (ICCs). The deviations (final model–initial model) of the AIC and BIC were −4655.00 and −4795.32, respectively, indicating an overall improvement in model fit. Next, we tested a bifactor structure with a general factor and two group factors, which showed adequate fit indices (RMSEA = 0.054, 95% CI [0.042–0.065], CFI = 0.988, TLI = 0.986) and a predominance of the general factor with a reasonable loading of all items (>0.40). The ωh coefficient for the general factor was 0.88, and those for the first and second group factors were 0.13 and 0.20, respectively. The percentage of ECV attributable to the general factor was 82.0%, while the remaining 18.0% was attributable to the group factors (10.7% and 7.3% attributable to the first and second group factors, respectively). All items had higher factor loadings on the general factor than on the group factors, indicating that the items predominantly reflected the general factor. Taken together, these findings suggest that the PREMIUM-RD item bank reflects an essentially unidimensional construct. Cronbach’s alpha was 0.94, and no residual correlation was greater than 0.20. Consequently, all 21 items in the PREMIUM-RD item bank met the requirements for IRT modeling and were kept for further analysis.

### 3.4. Calibration and Fitting of an IRT Model to the Data

The partial credit model (PCM) and the generalized partial credit model (GPCM) were used to calibrate the 21 items of the PREMIUM-RD item bank. The fit indices of the PCM were less adequate than those of the GPCM (14,546.64 and 14,249.58 for the AIC and 14,757.11 and 14,542.59 for the BIC, respectively), and the likelihood ratio test indicated a better fit of the GPCM compared with the PCM, X^2^ = 337.06, *p* < 0.001. As a result, we decided to use the GPCM to calibrate the PREMIUM-RD item bank. All items showed an adequate fit to the GPCM with respect to infit values ranging from 0.74 (item RD24) to 1.03 (item RD11). The discrimination parameters ranged from 0.68 (item RD7) to 3.35 (item RD1), and the threshold parameters ranged from −2.21 (item RD6) to 0.66 (item RD3) (Appendix A). Taken together, these results demonstrated that all items had moderate to very high discriminative power and that the threshold parameters reflected a broad spectrum of the latent trait, although there were relatively few items at the upper end of the continuum.

**Figure 1:**
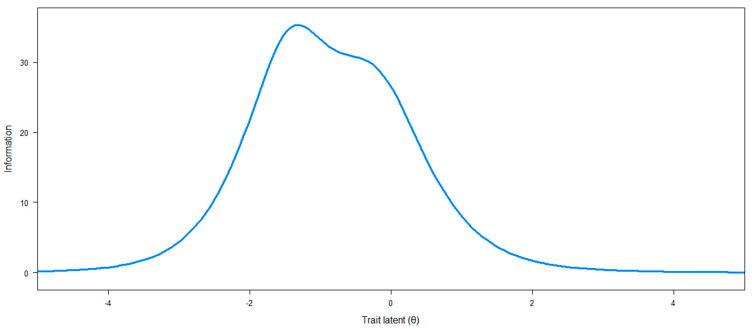
Test information.

The test information curve of the final PREMIUM-RD item bank is provided in Figure 1 and shows that the items have a high measurement precision over a broad spectrum of the latent trait (78.7% of total information is included in the [−2, 1] range of the latent continuum values) and that this precision is lower only for patients at the extremes, especially at the upper extreme (i.e., above 1). Item 19 was the most informative of the bank (“You felt confident”), whereas item 7 was the least informative (“You were embarrassed to have to answer intrusive questions”).

### 3.5. Evaluation of Differential Item Functioning (DIF)

Of the 84 tests performed (21 items with 4 confounding factors), 6 exhibited overall DIF. Following Zumbo’s DIF classification, no items were flagged for moderate or large DIF magnitudes, and only a few items were flagged for negligible DIF magnitudes: 1 item for diagnosis (item RD11) and 5 items for care setting (items RD1, RD2, RD12, RD20, and RD22). Given the negligible impact of these DIFs on the estimation of experience of respect and dignity, no items were deleted from the item bank (Appendix B).

### 3.6. External Validity of the Item Bank

The mean PREMIUM-RD score was 55.40 ± 22.43. Age was weakly correlated with PREMIUM-RD scores, and PREMIUM-RD scores were significantly higher for women and for non-single individuals. No significant differences were found by educational level, employment status, and care setting. Additionally, PREMIUM-RD scores were weakly correlated with GAF scores, and no correlation was found with duration of illness. PREMIUM-RD scores were significantly different according to the main diagnosis, with the highest scores observed for individuals with major depressive disorder and the lowest scores observed for patients with schizophrenia. PREMIUM-RD scores were strongly correlated with scores on the item measuring overall satisfaction with respect and dignity and the corresponding VAS. Finally, scores were weakly correlated with scores on SF-12 dimensions measuring physical functioning (PF), social functioning (SF), role physical (RP), role emotional (RE), vitality (VT), general health (GH), and composite scores of physical quality of life (PCS) and mental quality of life (MCS). Conversely, no correlation was observed between the dimensions of mental health (MH) and bodily pain (BP). The results regarding the external validation of the PREMIUM-RD item bank are presented in Table 3.

### 3.7. Elaboration of Item Administration Algorithm

Among the 3 scenarios tested, the CAT simulation with a level of precision of SEM < 0.33 was the most efficient, having the highest levels of accuracy (r = 0.97) and precision (RMSE = 0.23) while administering less than half of the items of the PREMIUM-RD item bank (on average 9 items). The other 2 simulations were not satisfactory, with a level of precision lower than expected (0.34 and 0.38, respectively), despite an adequate level of accuracy (r = 0.94 and r = 0.92, respectively) and a smaller average number of items administered (6 and 4 items, respectively). Table 4 provides the results of the CAT simulations.

## 4. Discussion

This work is part of the French PREMIUM initiative [22], which aims to provide a common measurement system for adult patients’ experience of care for three targeted conditions (including schizophrenia, bipolar disorder, or major depressive disorder) and is applicable in several care settings (i.e., outpatient and inpatient) based on item banks and CATs. In this article, we present the calibration of the PREMIUM-RD item bank and the development of the associated CAT, which captures all important aspects of the patients’ experience of respect and dignity during a hospital stay or consultation. Other item banks and associated CATs are under development by the PREMIUM project.

The final 21-item PREMIUM-RD item bank demonstrated strong psychometric properties (Appendix C). In particular, the assumptions required for IRT modeling (unidimensionality, local independence, and monotonicity) were fulfilled, and the GPCM showed an adequate fit to the data. The few indications of DIF were of negligible magnitude according to sex, age, care setting, and diagnosis. The item bank provides good information for a wide range of the latent continuum, although it may lack precision for patients at the highest extreme (i.e., for patients with high experience of respect and dignity). However, given that the goal of the measure is to identify aspects of patient experience that are suboptimal and therefore need to be improved, items that accurately distinguish patients with high experience of respect and dignity would be of limited interest; such items could be added at a later date if necessary. Additionally, this study provided preliminary evidence of the external validity of the PREMIUM-RD item bank. In particular, PREMIUM-RD scores were weakly correlated with GAF scores and SF-12 dimension scores, which is consistent with previous research that has shown a positive but weak association between patients’ experience and their outcomes [62,63]. In other words, PREMs provide important information for improving quality of care by identifying areas where change is needed, but these measures must be complemented by patient-reported outcome measures (PROMs) to provide a complete picture of quality of care from the patient’s perspective and support a patient-centered approach to care.

In addition to standard clinical indicators, patient experience is considered to be a valuable indicator of the quality of health care [64,65], and the use of PREMs is recommended by many organizations worldwide [66,67], but these data are not systematically collected in clinical psychiatric practice. In particular, organizational barriers, including a lack of time or resources to collect and analyze the data, have been reported in the literature [68]. The use of new technologies, based on item banks and CATs, has the potential to improve the use of these measures in routine clinical practice [19,20,28]. The PREMIUM-RD-CAT is the first adaptive PREM specific to adult patients with SMIs (i.e., schizophrenia, bipolar disorder, and major depressive disorder), which, unlike standard fixed-length questionnaires, administers only the most relevant items to the respondent, thereby reducing questionnaire completion time, increasing precision and providing a real-time score, thus minimizing patient and provider burden. The PREMIUM-RD-CAT, based on a level of precision of SEM < 0.33, showed adequate precision and accuracy, with correlations greater than 0.90 with scores based on the full bank of items and an RMSE less than 0.30. The validity of the PREMIUM-RD-CAT and its acceptability by stakeholders will be evaluated further in future analyses.

### 4.1. Implications for Clinical Practice

The use of a digital platform has great potential to improve the quality of mental health care. All adult patients with SMI will have the opportunity to complete a questionnaire after a hospital stay or consultation. Collecting data directly from patients will limit potential response bias and improve representativeness. This real-time feedback to mental health professionals has the potential to strongly improve the quality of care and decrease financial and human costs by optimizing patients’ care adherence, continuity of care, and improving health outcomes [63,69]. A digital platform will also allow for early identification of patients at risk of relapse and prevent potential care disruption, especially in the context of the COVID-19 pandemic. Finally, the digital platform will provide aggregated data for benchmarking within and across healthcare facilities and/or services. The financial allocation of psychiatry is currently based on activity, on the one hand, and on socio-demographic population data on the other hand [70]. In the future, funding could be modulated by the results of patients’ experience, within the framework of a quality-based financial allocation (IFAQ program—‘Incitation financière à l’amélioration de la qualité’—Financial Incentive to Quality Improvement [67].

### 4.2. Limitations

First, our sample size can be discussed. However, the sample size was sufficiently large to calibrate the item pool [71,72], and the sample included a diverse patient population, both inpatient and outpatient, from several facilities in different geographic regions of the country. Future work with a larger sample will improve the generalizability of the PREMIUM-RD item bank. Second, the selection of the IRT model could be discussed. In this study, we used the GPCM, although other models could have been used. Like the EORTC initiative [73], we used the GPCM rather than the GRM, both of which tend to yield similar results and should be viewed rather as alternatives than as competitors [74,75]. Additionally, given that the PCM is nested within the GPCM (in the PCM, all items have the same slope), their fit to the data can be compared. The GPCM generally provides a better fit to the data than a more parsimonious model such as the PCM. Third, the construct validity of the PREMIUM-RD item bank was assessed by examining relationships with GAF scores and SF-12 dimension scores. A high rate of missing data may have impacted the validity of our results. However, our results were consistent with our underlying assumptions. Additional work should be conducted to further assess the external validity of the PREMIUM-RD item bank. Finally, in this study, all items were administered to participants as part of a complete item bank, which is different from the administration of the CAT. Because the assessment of the precision and accuracy of the scores was based on the data used to calibrate the IRT model, these indicators may have been overestimated. Future work should assess the precision, accuracy, and validity of the PREMIUM-RD scores based on adaptive item administration using an independent sample.

## 5. Conclusions

The PREMIUM project aims to develop item banks of PREMs and CAT specific to SMIs (including schizophrenia, bipolar disorder, and major depressive disorder). This work reported satisfactory psychometric characteristics for respect and dignity measured using the PREMIUM-RD item bank, and the associated CAT showed a satisfactory level of precision, allowing for more accurate and flexible measurement of patient experience than that achieved by standard questionnaires. The use of advances in psychometric modeling and computer technologies will help improve the use of patient-reported measures in routine practice, thereby promoting a culture of patient-centered care.

## Figures and Tables

**Table 1:** Sample description.

	N(%) or Mean ± Standard Deviation		
	Total (n = 458)	Men (n = 280)	Women (n = 178)
**So** **cio-demographic data**			
Age, years (M ± SD) (n = 455)	38.11 ± 11.97	35.95 ± 10.88	41.51 ± 12.82
Marital status (single) (n = 373)	285 (76.4)	181 (84.2)	104 (65.8)
Educational level (<bachelor’s degree) (n = 374)	119 (31.8)	74 (34.6)	45 (28.1)
Employment status (unemployed) (n = 443)	331 (74.7)	192 (71.9)	139 (79.0)
**Clinical data**			
Care setting (n = 458)			
Outpatient	384 (83.8)	252 (90.0)	132 (74.2)
Inpatient	74 (16.2)	28 (10.0)	46 (25.8)
Under constraint	22 (29.7)	12 (42.8)	10 (21.7)
Main diagnosis (n = 456)			
Schizophrenia	287 (64.8)	222 (81.0)	65 (38.5)
Bipolar disorder	88 (19.9)	29 (10.6)	59 (34.9)
Major depressive disorder	68 (15.3)	23 (8.4)	45 (26.6)
Duration of illness, years (M ± SD) (n = 421)	12.34 ± 8.59	12.07 ± 7.94	12.74 ± 9.50
Global functionning (GAF score) (M ± SD) (n = 327)	57.07 ± 16.84	54.62 ± 15.70	60.98 ± 17.89
Quality of life (SF-12 scores) (M ± SD)			
PF (n = 270)	46.03 ± 11.66	48.11 ± 0.26	43.65 ± 2.71
SF (n = 271)	34.58 ± 11.87	36.44 ± 11.79	32.45 ± 11.63
RP (n = 271)	40.69 ± 11.04	41.77 ± 10.48	39.44 ± 11.57
RE (n = 270)	33.75 ± 12.31	35.08 ± 11.57	32.26 ± 12.97
MH (n = 272)	46.46 ± 8.37	46.71 ± 8.41	46.16 ± 8.34
VT (n = 271)	59.85 ± 12.06	58.09 ± 12.02	61.85 ± 11.84
BP (n = 272)	36.65 ± 14.47	36.78 ± 14.95	36.50 ± 13.97
GH (n = 271)	36.21 ± 11.49	35.00 ± 10.49	37.60 ± 12.44
PCS (n = 266)	41.72 ± 8.06	42.37 ± 8.38	40.97 ± 7.64
MCS (n = 266)	42.92 ± 9.72	43.00 ± 9.40	4282 ± 10.12

**Notes:** for each variable, the number of valid data is indicated. **Abbreviations**: *GAF* global assessment of functioning; *SF-12* medical outcome study 12-item Short Form, *PF* physical functioning, *SF* social functioning, *RP* role physical, *RE* role emotional, *MH* mental health, *VT* vitality, *BP* bodily pain, *GH* general health, *PCS* physical composite quality of life score, *MCS* mental composite quality of life score.

**Table 2:** Descriptive statistics of the PREMIUM-RD item bank.

Item No.	Item Content	Mean ± Standard Deviation	Floor Effect (%)	Ceiling Effect (%)	Missing Values (%)	Skewness Coefficient
**RD1**	You appreciated the welcome you received	3.40 ± 0.93	2.8	59.2	0.9	−1.96
**RD2**	Medical secrecy and the confidentiality of your information have been respected	3.36 ± 0.95	2.6	57.4	2.4	−1.77
**RD3**	You had easy access to the information in your medical record	2.36 ± 1.26	7.0	15.1	30.8	−0.35
**RD4**	Your bodily privacy has been respected	3.50 ± 0.82	1.5	60.0	5.5	−2.15
**RD5**	Your privacy has been respected	3.43 ± 0.86	1.7	59.0	1.7	−1.89
**RD6**	Your cultural and religious practices (beliefs, lifestyle, diet, etc.) have been respected	3.48 ± 0.74	0.4	43.4	26.0	−1.66
**RD7**	You were embarrassed to have to answer intrusive questions *	2.65 ± 1.38	9.0	35.6	7.4	−0.62
**RD8**	You have been the subject of hurtful remarks (about your physical appearance, your behavior, etc.) *	3.26 ± 1.11	4.1	54.6	5.9	−1.59
**RD9**	Some professionals have spoken in front of you as if you were not there *	3.14 ± 1.16	4.8	49.6	5.5	−1.37
**RD10**	You felt that you were not “taken seriously” *	2.92 ± 1.32	7.2	47.2	3.3	−0.96
**RD11**	You felt that the time spent with you was sufficient	2.79 ± 1.25	6.3	35.8	1.7	−0.83
**RD12**	You have felt negatively judged (“stigmatized”) *	3.07 ± 1.17	3.7	49.1	2.8	−1.11
**RD13**	You have been treated as a “whole person”	3.24 ± 1.00	3.1	49.8	1.3	−1.53
**RD14**	You felt like you were spoken to as an equal	2.97 ± 1.16	5.5	41.9	1.1	−1.06
**RD15**	You felt listened to	3.22 ± 1.02	3.3	51.5	0.0	−1.42
**RD16**	Your opinions have been taken into account	3.09 ± 1.08	4.6	43.7	1.3	−1.28
**RD17**	Your needs have been taken into account	3.09 ± 1.08	3.9	44.3	1.3	−1.22
**RD18**	Your rights have been respected	3.28 ± 0.93	2.2	49.3	1.1	−1.57
**RD19**	You felt confident	3.11 ± 1.06	3.7	45.4	1.3	−1.24
**RD20**	You think you have received all important information regarding your care	2.79 ± 1.18	5.9	31.9	2.2	−0.85
**RD21**	You think you have been involved in all important decisions regarding your care	2.84 ± 1.19	6.6	33.6	3.5	−0.99
**RD22**	You knew who to talk to when necessary	3.12 ± 1.02	3.1	43.0	2.2	−1.28
**RD23**	Your care has helped you to improve your well-being	3.02 ± 1.05	3.5	38.9	1.7	−1.09
**RD24**	Your care has met your expectations and needs	2.91 ± 1.07	3.3	33.8	2.4	−0.90

**Notes:** * items negatively worded and reverse scored for subsequent analyses.

**Table 3:** Comparison of PREMIUM-RD scores with socio-demographic and clinical data and proxy measures of quality of care.

	Correlation Coefficient (r)	Mean ± Standard Deviation	*p* Value
**Socio-demographic data**			
Age	0.18	-	<0.001
Sex	-		0.030
Men		53.59 ± 22.63	
Women		58.22 ± 21.28	
Marital status			<0.001
Single		53.47 ± 22.90	
Non-single		63.20 ± 21.60	
Educational level	-		0.852
<Bachelor’s degree		55.33 ± 23.23	
≥Bachelor’s degree		55.80 ± 22.44	
Employment status	-		0.053
Employed		59.10 ± 22.42	
Unemployed		54.38 ± 22.17	
**Clinical data**			
Care setting	-		0.825
Outpatient		55.29 ± 22.88	
Inpatient		55.93 ± 20.06	
Main diagnosis	-		<0.001
Schizophrenia		51.99 ± 21.77	
Bipolar disorder		58.89 ± 22.35	
Major depressive disorder		63.35 ± 20.73	
Duration of illness	−0.02	-	0.612
Global functioning (GAF score)	0.25	-	<0.001
**Proxy measures**			
Item of overall satisfaction	0.69	-	<0.001
VAS	0.72	-	<0.001
Quality of life (SF-12 scores)			
PF	0.14	-	0.027
SF	0.23	-	<0.001
RP	0.22	-	<0.001
RE	0.22	-	<0.001
MH	0.12	-	0.055
VT	0.23	-	<0.001
BP	−0.08	-	0.194
GH	0.20	-	<0.001
PCS	0.14	-	0.023
MCS	0.27	-	<0.001

**Abbreviations**: *GAF* global assessment of functioning; *VAS* visual analog scale; *SF-12* medical outcome study 12-items Short Form, *PF* physical functioning, *SF* social functioning, *RP* role physical; *RE* role emotional, *MH* mental health, *VT* vitality, *GH* general health, *PCS* physical composite quality of life score, *MCS* mental composite quality of life score.

**Table 4:** Mean scores and precision indicators for each CAT simulation.

Precision Level	Indicators	
**SEM < 0.33**	Mean score (±standard deviation)	56.41 ± 21.64
	Correlation coefficient (r)	0.97
	RMSE	0.23
	Mean number of items	8.49
**SEM < 0.44**	Mean score (±standard deviation)	52.08 ± 23.25
	Correlation coefficient (r)	0.94
	RMSE	0.34
	Mean number of items	5.60
**SEM < 0.55**	Mean score (±standard deviation)	52.01 ± 23.13
	Correlation coefficient (r)	0.92
	RMSE	0.38
	Mean number of items	4.11

**Abbreviations**: *SEM* standard error of measurement; *RMSE* root mean square error.

## Data Availability

The data are available on demand from the PREMIUM Scientific Committee.

## Appendix A

**Table A1:** Parameter estimates (discrimination and thresholds) and fit statistics for the 21 items in the final PREMIUM-RD item bank.

Item No.	Discrimination	Threshold 1	Threshold 2	Threshold 3	Threshold 4	Infit
**RD1**	3.35	−1.60	−0.40	-	-	0.92
**RD2**	1.78	−1.90	−0.42	-	-	0.88
**RD3**	0.81	−1.44	−1.26	−0.34	0.66	0.86
**RD4**	2.42	−1.90	−0.52	-	-	0.95
**RD6**	2.65	−2.21	−0.45	-	-	0.98
**RD7**	0.68	−0.80	0.05	-	-	0.89
**RD8**	1.40	−1.52	−0.51	-	-	0.96
**RD9**	1.58	−1.38	−0.27	-	-	0.84
**RD10**	1.79	−0.97	−0.19	-	-	0.74
**RD11**	1.13	−1.17	0.37			1.03
**RD12**	1.91	−1.25	−0.19	-	-	0.89
**RD13**	2.44	−1.61	−0.11	-	-	0.83
**RD14**	1.90	−1.36	−1.28	−0.90	0.03	0.95
**RD16**	2.95	−1.45	0.07	-	-	0.78
**RD18**	3.48	−1.56	−0.11	-	-	0.83
**RD19**	2.36	−1.53	−1.38	−1.01	−0.04	0.78
**RD20**	2.03	−1.23	0.51	-	-	0.81
**RD21**	2.10	−1.25	0.41	-	-	0.73
**RD22**	2.26	−1.58	0.10	-	-	0.84
**RD23**	1.79	−1.73	0.27	-	-	0.82
**RD24**	1.53	−1.84	−1.33	−0.95	0.37	0.74

## Appendix B

**Table A2:** DIF results.

Item No.	Sex		Age		Care Setting		Main Diagnosis	
	*p* Value	ΔR^2^	*p* Value	ΔR^2^	*p* Value	ΔR^2^	*p* Value	ΔR^2^
**RD1**	0.142	-	0.902	-	**0.001**	**0.018**	0.076	-
**RD2**	0.833	-	0.308	-	**<0.001**	**0.026**	0.356	-
**RD3**	0.669	-	0.047	-	0.479	-	0.749	-
**RD4**	0.668	-	0.152	-	0.219	-	0.412	-
**RD6**	0.578	-	0.560	-	0.075	-	0.661	-
**RD7**	0.057	-	0.548	-	0.153	-	0.325	-
**RD8**	0.443	-	0.468	-	0.062	-	0.758	-
**RD9**	0.076	-	0.096	-	0.085		0.777	-
**RD10**	0.126	-	0.131	-	0.451	-	0.955	-
**RD11**	0.885	-	0.692	-	0.481	-	**0.005**	**0.016**
**RD12**	0.405	-	0.152	-	**0.014**	**0.010**	0.182	-
**RD13**	0.224	-	0.299	-	0.076	-	0.709	-
**RD14**	0.800	-	0.727	-	0.048	-	0.639	-
**RD16**	0.534	-	0.700	-	0.098	-	0.870	-
**RD18**	0.109	-	0.280	-	0.016	-	0.137	-
**RD19**	0.145	-	0.073	-	0.876	-	0.204	-
**RD20**	0.242	-	0.230	-	**<0.001**	**0.020**	0.893	-
**RD21**	0.777	-	0.027	-	0.059	-	0.690	-
**RD22**	0.578	-	0.838	-	**0.003**	**0.014**	0.248	-
**RD23**	0.947	-	0.226	-	0.543	-	0.087	-
**RD24**	0.030	-	0.168	-	0.732	-	0.720	-

**Notes:** Bold values indicate DIF *p* value <0.01. ΔR^2^: DIF magnitude: negligible (ΔR^2^ < 0.13), moderate (0.13 ≤ ΔR^2^ ≥ 0.26), or large (ΔR^2^ ≥ 0.26).

## Appendix C

**Table A3:** List of the 21 items of the PREMIUM-RD item bank (English and French versions).

Items No.	Item Content in English	Item Content in French
**RD1**	You appreciated the welcome you received	Vous avez apprécié(e) la façon dont vous avez été accueilli(e)
**RD2**	Medical secrecy and the confidentiality of your information have been respected	Le secret médical et la confidentialité des informations vous concernant ont été respectés
**RD3**	You had easy access to the information in your medical record	Vous avez pu facilement avoir accès aux informations contenues dans votre dossier médical
**RD4**	Your bodily privacy has been respected	Votre intimité corporelle a été respectée
**RD6**	Your cultural and religious practices (beliefs, lifestyle, diet, etc.) have been respected	Vos pratiques culturelles et religieuses (croyances, habitudes de vie, alimentation, etc.) ont été respectées
**RD7**	You were embarrassed to have to answer intrusive questions	Vous avez été gêné(e) d’avoir à répondre à des questions indiscrètes
**RD8**	You have been the subject of hurtful remarks (about your physical appearance, your behavior, etc.)	Vous avez fait l’objet de remarques blessantes (sur votre apparence physique, votre comportement, etc.)
**RD9**	Some professionals have spoken in front of you as if you were not there	Certains professionnels ont parlé devant vous comme si vous n’étiez pas là
**RD10**	You felt that you were not “taken seriously”	Vous avez l’impression de ne pas « être pris(e) au sérieux »
**RD11**	You felt that the time spent with you was sufficient	Vous avez eu l’impression que le temps qui vous a été consacré était suffisant
**RD12**	You have felt negatively judged (“stigmatized”)	Vous avez eu le sentiment d’être jugé négativement (« ressenti de la stigmatisation »)
**RD13**	You have been treated as a “whole person”	Vous avez été traité(e) comme un « individu à part entière »
**RD14**	You felt like you were spoken to as an equal	Vous avez ressenti que l’on vous parlait d’« égal à égal »
**RD16**	Your opinions have been taken into account	Vos opinions ont été prises en compte
**RD18**	Your rights have been respected	Vos droits ont été respectés
**RD19**	You felt confident	Vous vous êtes senti(e) en confiance
**RD20**	You think you have received all important information regarding your care	Vous pensez avoir reçu toutes les informations importantes sur votre prise en charge
**RD21**	You think you have been involved in all important decisions regarding your care	Vous pensez avoir été impliqué(e) dans les décisions importantes de votre prise en charge
**RD22**	You knew who to talk to when necessary	Vous avez su à qui vous adresser quand vous en avez eu besoin
**RD23**	Your care has helped you to improve your well-being	Votre prise en charge vous a aidé(e) à améliorer votre bien-être
**RD24**	Your care has met your expectations and needs	Votre prise en charge a répondu à vos attentes et vos besoins
